# Human inborn errors of immunity underlying *Talaromyces marneffei* infections: a multicenter, retrospective cohort study

**DOI:** 10.3389/fimmu.2025.1492000

**Published:** 2025-01-22

**Authors:** Huifeng Fan, Zhiyong Yang, Yuhui Wu, Xiulan Lu, Tian Li, Xuyang Lu, Gen Lu, Liming He, Guoping Lu, Li Huang

**Affiliations:** ^1^ Department of Respiratory Infection, Guangzhou Women and Children’s Medical Center, Guangzhou Medical University, Guangzhou, China; ^2^ Department of Pediatrics, The First Affiliated Hospital of Guangxi Medical University/Difficult and Critical Illness Center, Pediatric Clinical Medical Research Center of Guangxi, Nanning, China; ^3^ Pediatric Intensive Care Unit, Shenzhen Children’s Hospital, Shenzhen, Guangdong, China; ^4^ Department of Pediatrics, Hunan Children’s Hospital, Changsha, China; ^5^ Pediatric Intensive Care Unit, Guangzhou Women and Children’s Medical Center, Guangzhou Medical University, Guangzhou, China; ^6^ Pediatric Intensive Care Unit, Children’s Hospital of Fudan University, Shanghai, China; ^7^ Pediatric Emergency Department, Guangzhou Women and Children’s Medical Center, Guangzhou Medical University, Guangzhou, China

**Keywords:** *Talaromyces marneffei*, inborn errors of immunity, immune status, gene mutation, children

## Abstract

**Introduction:**

*Talaromyces marneffei* (*T. marneffei*) infections in children can occur secondary to inborn errors of immunity (IEIs). We aimed to investigate the clinical and genetic features of *T. marneffei* infection in Chinese pediatric patients.

**Materials and methods:**

We retrospectively reviewed 18 pediatric patients with IEIs who were diagnosed with *T. marneffei* infections at five public hospitals in China from January 2015 to January 2023.

**Results:**

The common clinical features among the patients were fever, cough, and hepatomegaly. The most common severe complications included septic shock, hemophagocytic lymphohistiocytosis (HLH), and acute respiratory distress syndrome (ARDS). Three cases presented with pan-hypogammaglobulinemia, while three other cases showed heightened levels of IgM. Elevated levels of IgE were detected in five cases, and six cases exhibited decreased T lymphocyte absolute counts. Four children were diagnosed with hyperimmunoglobulin M syndrome (HIGM) due to *CD40LG* mutations, three cases had severe combined immunodeficiency (SCID), and five were diagnosed with hyper-IgE syndrome (HIES). Gain-of-function (GOF) mutations in *STAT1* led to STAT1 GOF in four cases. One patient was diagnosed with caspase-recruitment domain (CARD9) deficiency due to a compound mutation in the *CARD9* gene, while another patient was confirmed with adenosine deaminase (ADA) deficiency.

**Conclusion:**

*T. marneffei* infections in children with IEIs induced severe systemic complications. These children commonly exhibited abnormal immunoglobulin levels in peripheral blood, and underlying IEIs associated with *T. marneffei* infections have enhanced our understanding of the disease.

## 1 Introduction

*Talaromyces* (formerly *Penicillium*) *marneffei* (*T. marneffei*) is a thermally dimorphic fungus endemic to Southeast Asia that causes systemic infections in humans (1). This fungus can grow in a filamentous form at 25°C–30°C or in a yeast-like form at 37°C (2), and it exists as yeast inside the host body (3). *T. marneffei* infection was first observed in bamboo rats in 1956 (4), and the first reported case of natural human infection occurred in 1973 (5). In adults, *T. marneffei* infection has been exclusively associated with acquired immunodeficiency syndrome (AIDS) caused by human immunodeficiency virus (HIV) infection (6). Notably, *T. marneffei* was ranked second among the world’s 10 most feared fungi in 2018 (7). In addition to the increasing *T. marneffei* infection rates among individuals with HIV, a rise in infections has also been observed in HIV-uninfected but immunocompromised patients (10.1%) since the mid-1990s (8). *T. marneffei* infection in HIV-uninfected children should be taken seriously, as it can lead to high mortality rates exceeding 50% in previous reports (9, 10), yet the symptoms and signs are often atypical (11). Therefore, identifying the underlying disease is a key link in the diagnosis and treatment of these patients.

In contrast to adults, pediatric patients with human inborn errors of immunity (IEIs) are more susceptible to *T. marneffei* infections (9, 12). The prevalence of *T. marneffei* infection is particularly high in HIV-uninfected pediatric patients with severe combined immunodeficiencies (SCID), hyperimmunoglobulin (hyper-IgE) syndrome (HIES), *CD40LG* deficiency, and others (13–15). We review the IEIs known to predispose individuals to *T. marneffei* infections. Understanding the pathogenesis of *T. marneffei* infections in HIV-uninfected patients is crucial. Additionally, it is also timely to decipher the cellular and molecular mechanisms of antifungal immunity while developing new approaches for treating *T. marneffei* infections.

## 2 Materials and methods

### 2.1 Study design and population

A retrospective cohort study was conducted from January 2015 to January 2023 at five public hospitals in China. The inclusion criteria for the study were as follows (1): 28 days < age ≤ 18 years; (2) culture or histopathologically proven infections caused by *T. marneffei*; and (3) diagnosed IEIs. Exclusion criteria were HIV infection, cancer therapy, and organ transplantation.

### 2.2 Diagnosis of *T. marneffei* infections

The diagnosis of *T. marneffei* infections included a positive culture of *T. marneffei* from blood, bone marrow, and other clinical specimens on Sabouraud dextrose agar, following standard culture techniques. Identification was based on the morphology of the colonies. *T. marneffei* grew as a mold form at 25℃ and a yeast form at 37℃. At 25℃, it produced a soluble red pigment that diffused into the agar. Under the microscope, a typical broomstick shape with septal hyphae can be observed.

### 2.3 Diagnosis of IEIs

The diagnosis of IEIs was based on clinical characteristics and genetic tests, according to the updated classification by the Human Inborn Errors of Immunity Committee of the International Union of Immunological Societies (IUIS) (16). The predicted pathogenicity of novel variants was evaluated based on the American College of Medical Genetics and Genomics (ACMG) and the Association for Molecular Pathology (AMP) criteria (17). As noted, the National Institutes of Health (NIH) developed a clinical hyperimmunoglobulin E syndrome (HIES) scoring system (18), which can serve as a valuable reference for the diagnosis of HIES. For novel missense variants, minor allele frequency (MAF), combined annotation dependent depletion (CADD) score, and rare exome variant ensemble learner (REVEL) were used to evaluate pathogenicity.

### 2.4 Data collection

All data were collected using a standardized form based entirely on the medical reports of each patient. The data included demographic information, domiciles, medical history, clinical manifestations, immunologic detection, genetic tests, complications, and prognosis. For patients with multiple admissions, data from the first admission were collected.

### 2.5 Ethical statement

Ethical approval has been obtained from the National Key Research and Development Program of China (2021YFC2701801, 2021YFC2701803, 2021YFC2701805). All patients provided written informed consent for the use of their clinical and laboratory data from their medical reports.

## 3 Results

### 3.1 Clinical characteristics

A total of 18 children were enrolled in this study. The clinical features of the children are summarized in **Table 1**; **Supplementary Table S1**. There were 15 boys and 3 girls, with diagnostic ages ranging from 3 to 200 months (median age: 18.5 months). The median interval between onset and diagnosis was 0.7 months (interquartile range [IQR]: 0.33–2.0 months). The most common clinical presentations of *T. marniffei* infections were fever, cough, and hepatomegaly. Life-threatening complications during hospitalization included septic shock, acute respiratory distress syndrome (ARDS), hemophagocytic lymph histiocytosis (HLH), multiple organ dysfunction syndrome (MODS), and disseminated intravascular coagulation (DIC). Most patients (10/18, 55.56%) were confirmed by blood culture, with eight of them also confirmed by bone marrow specimens. In addition, two cases underwent airway mucosal biopsy. Antifungal therapy was administered in all cases, with a treatment course of 23 weeks (IQR: 12–26 weeks). Voriconazole was the most frequently used antifungal agent (10/18, 55.56%). The median length of stay was 25.5 days. However, three children (3/18, 16.67%) eventually died from *T. marniffei* infections (P8, P13, and P16), with recorded ages at death of 3 months, 4 months, and 17 months, respectively.

**Table 1:** Clinical characteristics of children with IEI with *T. marneffei*.

Clinical characteristics	Cases (No = 18)
Demographics	
Age (months; median [IQR])	18.5 (12.25–46)
Sex (male [%])	15 (83.33)
The interval between onset and diagnosis (months; median [IQR])	0.7 (0.325–2)
Signs and symptoms	
Fever (No. [%])	16 (88.89)
Cough (No. [%])	15 (83.33)
Hepatomegaly (No. [%])	13 (72.22)
Splenomegaly (No. [%])	12 (66.67)
Lymphadenopathy (No. [%])	11 (61.11)
Weight loss (No. [%])	10(55.56)
Malnutrition (No. [%])	9 (50.00)
Diarrhea (No. [%])	8(44.44)
Skin lesion (No. [%])	6 (33.33)
Dyspnea (No. [%])	6 (33.33)
Ascites (No. [%])	1 (5.56)
Trachyphonia (No. [%])	1 (5.56)
Significant complication	
Total (No. [%])	12 (66.67)
Sepsis shock (No. (%)	8 (44.44)
HLH (No. [%])	4 (22.22)
ARDS (No. [%])	7 (38.89)
MODS (No. [%])	3 (16.67)
DIC (No. [%])	2 (11.11)
Specimens for diagnosis	
Blood (No. [%])	10 (55.56)
BM (No. (%)	8 (38.10)
Sputum (No. [%])	6 (33.33)
BALF (No. [%])	3 (16.67)
Lymph nodes (No. [%])	2 (11.11)
Ascites (No. [%])	1 (5.56)
Airway mucosal biopsy (No. [%])	1 (5.56)
Lung biopsy (No. [%])	1 (5.56)
Stool (No. [%])	1 (5.56)
Antifungal therapy	
Total (No. [%])	18 (100.00)
Amphotericin B (No. [%])	7 (38.89)
Voriconazole (No. [%])	10 (55.56)
Itraconazole (No. [%])	8 (38.10)
Micafungin (No. [%])	2 (11.11)
Caspofungin (No. [%])	1 (5.56)
**The course of antifungal therapy (weeks; median [IQR])**	23 (12–26)
Outcomes	
Length of stay (days; median [IQR])	25.5 (14.25–32)
Mortality (No. [%])	3 (16.67)

ARDS, acute respiratory distress syndrome; MODS, multiple organ dysfunction syndrome; HLH, hemophagocytic lymphohistiocytosis; DIC, disseminated intravascular coagulation; BM, bone marrow; BALF, bronchial alveolar lavage fluid; IQR, interquartile range.

### 3.2 Peripheral immunological evaluation

The immunologic detection and genetic tests at the time of diagnosis are shown in **Table 2**; **Supplementary Table S2**. All patients were HIV-negative, as determined by a serum-specific antibody test. The lymphocyte count, immunoglobulin and complement levels, and nitroblue tetrazolium (NBT) test results in peripheral blood were detected in all cases. Among them, three cases presented with pan-hypogammaglobulinemia (3/18, 16.67%) (P5, P12, and P13), three cases had heightened levels of IgM (3/18, 16.67%) (P6, P9, and P15), and five cases showed higher levels of IgE (5/18, 27.78%) (P1, P4, P9, P10, and P15). Low complement C3 levels were found in three cases (P8, P11, and P12). Six cases presented decreasing T lymphocyte counts, including CD 4+ and CD 8+ subsets in the results (P3, P8, P12, P13, P16, and P17). More than half of all cases (P3, P8, P9, P10, P12, P13, P14, P16, P17, and P18) had markedly decreased NK cell counts (10/18, 55.56%). The inverted CD4/CD8 ratio was observed in four patients (P4, P10, P12, and P13).

**Table 2:** Peripheral immunological evaluation of children with IEIs infected by *T. marneffei*.

Laboratory assays of immunity	Cases (No = 18)
Immunoglobulin	
Pan-hypogammaglobulinemia (IgG, IgA, and IgM; No. [%])	3 (16.67)
IgG decrease (No. [%])	7 (38.89)
IgA decrease (No. [%])	6 (33.33)
IgE increase (No. [%])	5 (27.78)
IgM decrease (No. [%])	5 (27.78)
IgM increase (No. [%])	3 (16.67)
Complements	
C3 decrease (No. [%])	3 (16.67)
C4 decrease (No. [%])	0 (0.00)
Lymphocytes	
T lymphocyte counts decrease (CD3+; No. [%])	6 (33.33)
CD4+ subsets decrease (No. [%])	6 (33.33)
CD19+ subsets decrease (No. [%])	5 (26.32)
Inverted CD4/CD8 ratio (No. [%])	4 (22.22)
NK cells decrease (No. [%])	10 (55.56)
Neutrophils	
Neutropenia (No. [%])	11 (61.11)
Abnormal human neutrophil respiratory burst (No. [%])	0 (0.00)

Ig, immunoglobulin; NK cells, natural killer cells; HIV, human immunodeficiency virus.

### 3.3 Genetic mutation and human inborn errors of immunity

In this cohort, eleven patients underwent gene panel sequencing, and whole exome sequencing (WES) was performed in seven patients. A total of 11 novel variants were identified in nine patients. According to the ACMG/AMP criteria, three novel variants were classified as likely pathogenic, and the rest were classified as pathogenic (**Table 3**). MAF, CADD score, and REVEL analyses were performed for four novel missense variants, and three variants were found to be harmful (P10, P15, and P16) (**Supplementary Table S3**). Four children (P5, P6, P7, and P14) were confirmed to have CD40 ligand deficiency due to mutations or microdeletion in the *CD40LG* gene. Three patients (P8, P12, and P13) were diagnosed with SCID caused by *IL2RG* mutations. Five cases were diagnosed with autosomal dominant-hyper immunoglobulin E syndrome (AD-HIES) due to mutations in *STAT3*, with a NIH score greater than 40 points for the novel *STAT3* variants (**Supplementary Table S4**). Gain-of-function (GOF) mutations in *STAT1* resulted in STAT1 GOF in four cases (P2, P3, P17 and P18). P11 was diagnosed with caspase-recruitment domain 9 (CARD9) deficiency due to compound mutations in the *CARD9* gene, while P16 was confirmed to have adenosine deaminase (ADA) deficiency resulting from compound mutations in the *ADA* gene.

**Table 3:** Genetic mutations and human inborn errors of immunity in children with *T. marneffei*.

Patient	Genetic locus	Nucleotide variation	Types of gene mutation	Mutation source	Protein consequence	IEIs	Inheritance	Previously reported	ClinVar	Pathogenicity (ACMG classification)
P1	*STAT3*	c.1859C>G	Missense mutation	Sporadic	p.Thr620Ser	AD-HIES Job syndrome	AD LOF	Yes (33, 34)	Not found	Pathogenic, PS1, PS2, PM2, PP2, PP3, PP4
P2	*STAT1*	c.520T>C	Missense mutation	Sporadic	p.Cys174Arg	STAT1 GOF	AD GOF	Yes (31, 35, 36)	Not found	Pathogenic, PS1, PS2, PM2, PP2, PP3, PP4
P3	*STAT1*	c.1154C>T	Missense mutation	Sporadic	p.Thr385Met	STAT1 GOF	AD GOF	Yes (37–39)	Not found	Pathogenic, PS1, PS2, PM2, PP2, PP3, PP4
P4	*STAT3*	c.1673G>A	Missense mutation	Sporadic	p.Gly558Asp	AD-HIES Job syndrome	AD LOF	Yes (40, 41)	Not found	Pathogenic, PS1, PS2, PM2, PP2, PP3, PP4
P5	*CD40LG*	c.424_436del	Frameshift mutation	Maternal	p.Glu142Thrfs*3	CD40 ligand deficiency (CD154)	XL	No	Not found	Pathogenic, PVS1, PM2, PP3, PP4
P6	*CD40LG*	> 132 kb	Fragment deletion	Maternal	–	CD40 ligand deficiency (CD154)	XL	No	Not found	Pathogenic, PVS1, PM2, PM4, PP4
P7	*CD40LG*	c.598A>T	Nonsense mutation	Maternal	p.Arg200Ter	CD40 ligand deficiency (CD154)	XL	Yes (42, 43)	Not found	Pathogenic, PVS1, PM2, PS1, PP4
P8	*IL2RG*	c.185G>A	Missense mutation	Maternal	p.Cys62Tyr	gc deficiency (common gamma chain SCID, CD132 deficiency)	XL	Yes (44)	Not found	Pathogenic, PS1, PM2, PM4, PP2, PP3, PP4
P9	*STAT3*	c.1679-1681del	Frameshift mutation	Sporadic	p.Ser560del	AD-HIES Job syndrome	AD LOF	No	Not found	Pathogenic, PS2, PM2, PM5, PP2, PP3, PP4
P10	*STAT3*	c.1593A>T	Missense mutation	Sporadic	p.Lys531Asn	AD-HIES Job syndrome	AD LOF	No	Not found	Pathogenic, PS1, PS2, PM2, PP2, PP3, PP4
P11	*CARD9*	c.1118G>C	Missense mutation	Maternal	p.Arg373Pro	CARD9 deficiency	AR	No	Not found	Likely pathogenic, PM2, PM3, PP4
		Exon2-19del	Nonsense mutation	Paternal	–			No	Not found	Pathogenic, PVS1, PM2, PM3, PP4
P12	*IL2RG*	c.464G>A	Nonsense mutation	Maternal	p.Trp155X	gc deficiency (common gamma chain SCID, CD132 deficiency)	XL	No	Not found	Pathogenic, PVS1, PS4, PM2, PP2, PP3, PP4
P13	*IL2RG*	c.464G>A	Nonsense mutation	Maternal	p.Trp155X	gc deficiency (common gamma chain SCID, CD132 deficiency)	XL	No	Not found	Pathogenic, PVS1, PS4, PM2, PP2, PP3, PP4
P14	*CD40LG*	c.1978 + 1G>A	Shear mutation	Maternal	–	CD40 ligand deficiency (CD154)	XL	Yes (43)	Not found	Pathogenic, PVS1, PS1, PM4, PP4
P15	*STAT3*	c.115G>A	Missense mutation	Sporadic	p.Glu39Lys	AD-HIES Job syndrome	AD LOF	No	Not found	Likely pathogenic, PS2, PM2, PP3, PP4
P16	*ADA*	c.730delG	Frameshift mutation	Maternal	p.Glu244Lysfs*67	Adenosine deaminase (ADA) deficiency	AR	No	Not found	Pathogenic, PVS1, PM2, PP3, PP4
		c.202T>A	Missense mutation	Paternal	p.Tyr68Asn			No	Not found	Likely pathogenic, PM2, PM3, PP4
P17	*STAT1*	c.1170G>A	Missense mutation	Sporadic	p.Met390Ile	STAT1 GOF	AD GOF	Yes (20, 40, 45)	Not found	Pathogenic, PS2, PS4, PM1, PM2, PM5, PP2, PP3, PP4
P18	*STAT1*	c.1053G>T	Missense mutation	Sporadic	p.Leu351Phe	STAT1 GOF	AD GOF	Yes (46, 47)	Not found	Pathogenic, PS1, PS2, PM2, PP2, PP3, PP4

STAT, signal transducers and activators of transcription; CARD, caspase-recruitment domain; IL, interleukin; ADA, adenosine deaminase deficiency; SCID, severe combined immune deficiency; AD, autosomal dominant; LOF, loss-of-function; GOF, gain-of-function; XL, X-linked; AR, autosomal recessive; PVS, pathogenic very strong; PS, pathogenic strong; PM, pathogenic moderate; PP, pathogenic supporting.

## 4 Discussion

*T. marneffei* is a saprophytic pathogenic fungus capable of causing fatal systemic mycosis in immunocompromised hosts, mostly in tropical and subtropical Asia (19). In adults, *T. marneffei* infection predominantly affects AIDS patients, where it manifests as a severe deep mycosis with high mortality (6). With advancements in clinical immunological diagnostics, children with IEIs have been documented as vulnerable to *T. marneffei* infection (9, 12). Conditions such as *CD40L* deficiency, autosomal dominant (AD) hyper-IgE syndrome, IL-12/IFN-γ axis deficiency, and other unknown specific immune defects are increasingly associated with pediatric talaromycosis in HIV-uninfected patients (20). Furthermore, recognizing IEIs underlying *T. marneffei* infections is important to reduce mortality in children and facilitate the investigation of the pathogenic mechanism of *T. marneffei* infections. We retrospectively analyzed pediatric patients with *T. marneffei* infections between January 2015 and January 2023 at five public hospitals in China, all of them had diagnosed cases of IEIs through genetic testing. We summarized the clinical characteristics and peripheral immunity status of 18 children with *T. marneffei* infections over this decade. Meanwhile, we demonstrated diverse IEIs involving *T. marneffei* infections, which provides valuable insights into the pathogenic mechanisms of *T. marneffei* infections.

In pediatric patients with IEIs, the clinical manifestations of *T. marneffei* infections are more complex and severe than in HIV-infected patients (21). In this cohort, most cases presented with fever, cough, hepatosplenomegaly, lymphadenopathy, and weight loss simultaneously. Nonspecific clinical manifestations can potentially lead to the misdiagnosis of *T. marneffei* infection in HIV-uninfected children. However, serious complications such as HLH, septic shock, MODS, DIC, and ARDS were observed in some cases, as previously reported (22). In the present study, three fatalities were observed in children under the age of 2 years, with two cases involving infants aged 3 and 4 months, respectively. Despite rapid diagnosis, these infants developed serious complications and eventually died. Prior research indicated that 36.0% of pediatric patients experienced severe complications (23); however, in our study, the incidence was notably higher at 66.67%. It is important to highlight that systematic complications associated with IEIs in pediatric patients are prevalent and multifaceted. Pediatricians are made aware of this concern to facilitate the identification of *T. marneffei* infections and to prevent mortality.

In the present study, all these children were diagnosed with IEIs, and as a result, they exhibited abnormal immune parameters at the time of diagnosis, to varying degrees, including decreased levels of IgG and increased levels of IgE in peripheral blood. However, only in five cases did the number of peripheral blood lymphocytes significantly decreased. Previous studies have suggested that a reduction in the number of T lymphocytes or cellular immunity is probably the most important predisposing factor for *T. marneffei* infection in HIV-infected patients (9, 21, 24). Unlike HIV-infected patients, the common immunological findings in IEI children with *T. marneffei* infections are abnormal immunoglobulin changes. Meanwhile, reduced NK cell counts were identified in most patients. NK cells are the prototype innate lymphoid cells endowed with potent cytolytic functions that provide host defense against microbial infections (25). Previous research has indicated that NK cells are frequently found in HIV-negative *T. marneffei*-infected patients (20, 26). NK cells might play an important role in defense against *T. marneffei.* Further research is required to explore the mechanisms by which NK cells respond to *T. marneffei* infections.

After encountering *T. marneffei*, the host depends on both innate and acquired immune responses to eradicate the microorganism and combat infection. Multiple studies have shown that depletions of CD4 T lymphocytes is closely associated with an increased risk of *T. marneffei* infections (9, 27). Immune deficiencies involving cellular-mediated immune responses and CD4 lymphopenia have been documented to be associated with *T. marneffei* infection, thus increasing susceptibility to the infection (14, 27, 28). CD4 T-cell-mediated immunity, mainly Th1 and Th17 responses, is essential for protection against dimorphic fungi. In the gene interaction network of the cohort, *IL2RG* was the capital gene for typical X-SCID. ADA, a purine salvage pathway deficiency, results in a buildup of toxic metabolites, causing death in rapidly dividing cells, especially lymphocytes. The most complete form of ADA also leads to SCID (29). In this cohort, four patients were diagnosed with SCID, including three cases of X-SCID and one case of ADA deficiency. Among them, two infants of the three recorded fatalities were attributed to X-SCID resulting from mutations in the *IL2RG* gene, while the remaining case was associated with ADA deficiency, which is also classified as SCID. Only one X-SCID patient survived after antifungal therapy. Therefore, *T. marneffei* infections can be lethal in individuals with SCID.

The low proportions of circulating Th17 cells result from impaired pro-Th17 cytokine signaling or production (e.g., STAT3-HIES, which impairs signaling downstream from IL-6, IL-23, and IL-21, in particular) (30); an increase in signaling downstream from cytokines that inhibit Th17 cell differentiation (e.g., STAT1 GOF, which increases cellular responses to IFNs and IL-27, both of which inhibit Th17 cell differentiation) (31); and the impaired production of pro-Th17 cytokines by phagocytes upon fungal recognition (e.g., CARD9 deficiency) (32). Therefore, STAT3-HIES, STAT1 GOF, and CARD9 deficiency had varying degrees of impact on the induction of Th17. It was indeed found that patients with these IEIs, leading to increased susceptibility to *T. marneffei* infections in the cohort, exhibited such an effect. An analysis of the molecular and cellular basis of *T. marneffei* infections in these IEIs has suggested a possible role of Th17-mediated immunity in protection against *T. marneffei* infections.

The study still has some limitations that should be considered. This is a multicenter retrospective analysis, and 18 cases of IEIs were confirmed by gene sequencing from five hospitals over the past 8 years. Some HIV-negative children with *T. marneffei* did not receive genetic tests, and hence the proportion of such cases with underlying IEIs is unknown. In the retrospective study, a total of 11 novel variants were identified in nine patients. Relevant functional testing will be performed in the future to verify the potential mechanisms of these novel variants. Nevertheless, this study may provide a valuable reference for underlying IEIs of *T. marneffei* infection in HIV-negative children. Additionally, we will continue to focus on *T. marneffei* infections in pediatric populations and pursue investigations into the pathogenic mechanisms associated with *T. marneffei* infection.

## 5 Conclusion

*T. marneffei* infections in children with IEIs often involve severe systemic complications, necessitating thorough and careful observation by clinicians for early identification. Significant immunoglobulin abnormalities were observed in the peripheral blood of these children with severe *T. marneffei* infections. The study of IEIs underlying *T. marneffei* infections has deepened our understanding of the condition.

## Data Availability

The original contributions presented in the study are included in the article/**Supplementary Material**. Further inquiries can be directed to the corresponding authors.
