# Supplementary material for: Human inborn errors of immunity underlying Talaromyces marneffei infections: a multicenter, retrospective cohort study
Source: Front Immunol. 2025 Jan 22;16:1492000. doi: 10.3389/fimmu.2025.1492000 (PMC11794527; doi:10.3389/fimmu.2025.1492000)
Supplement: Supplementary file 1 [file Table1.docx]

| **TABLE S1 Clinical manifestations of IEIs children with *T. marneffei*** | | | | | | | | | | | | | |
| --- | --- | --- | --- | --- | --- | --- | --- | --- | --- | --- | --- | --- | --- |
| Patients | Age of Diagnosis  (months) | Gender | District of China | The interval between onset and diagnosis  (days) | The interval between onset and diagnosis  (months) | Clinical manifestations | Complications | | Specimens for diagnosis | Antifungal therapy | The course of antifungal therapy  (weeks) | Length of stay  (days) | Prognosis |
| P1 | 37 | Male | Hunan | 3 | 0.1 | Fever, Diarrhea, Lymphadenopathy, Hepatosplenomegaly | Sepsis shock | | Sputum, Blood | Amphotericin B liposomes, Voriconazole | 6 | 28 | Improvement |
| P2 | 104 | Male | Hunan | 4380 | 48 | Fever, Cough, Weight loss, Skin lesion, Lymphadenopathy |  | | Bone marrow，Blood | Itraconazole | 26 | 7 | Improvement |
| P3 | 18 | Male | Guangxi | 60 | 2 | Fever, Cough, Lymphadenopathy, Hepatosplenomegaly | HLH | | Lymph nodes | Voriconazole | 24 | 68 | Improvement |
| P4 | 49 | Male | Guangxi | 93 | 3 | Fever, Diarrhea, Lymphadenopathy, Hepatosplenomegaly |  | | Bone marrow, Blood, Liver biopsy, Stool | Voriconazole | 24 | 23 | Improvement |
| P5 | 13 | Male | Guangdong | 10 | 0.3 | Fever, Cough, Malnutrition, Weight loss | ARDS, HLH, DIC, Sepsis shock, MODS | | Blood | Itraconazole | 28 | 67 | Improvement |
| P6 | 12 | Male | Guangdong | 15 | 0.5 | Fever, Cough, Skin lesion, Lymphadenopathy, Hepatosplenomegaly |  | | Blood | Voriconazole | 22 | 38 | Improvement |
| P7 | 19 | Male | Guangdong | 30 | 1 | Fever, Cough, Diarrhea, Lymphadenopathy, Hepatomegaly | ARDS | | Airway mucosal biopsy, Blood | Voriconazole | 26 | 22 | Improvement |
| P8 | 3 | Male | Guangdong | 7 | 0.3 | Fever, Diarrhea, Dyspnea, Skin lesion, Hepatosplenomegaly | ARDS, HLH, DIC, Sepsis shock, MODS | | Blood, Bone marrow | Caspofungin, Amphotericin B liposomes | 0.5 | 4 | Died |
| P9 | 19 | Male | Guangdong | 61 | 2 | Cough, Dyspnea | ARDS | | Sputum | Voriconazole, Itraconazole | 25 | 47 | Improvement |
| P10 | 156 | Male | Hainan | 20 | 0.7 | Cough, Dyspnea, Malnutrition, Skin lesion, Trachyphonia, Lymphadenopathy | ARDS | | Sputum | Amphotericin B liposomes, Voriconazole | 12 | 25 | Improvement |
| P11 | 12 | Female | Guangdong | 10 | 0.3 | Fever, Cough, Diarrhea, Malnutrition, Weight loss, Lymphadenopathy | Sepsis shock | | Sputum, Bone marrow | Amphotericin B liposomes, Itraconazole | 28 | 28 | Improvement |
| P12 | 8 | Male | Guangdong | 20 | 0.7 | Fever, Cough, Diarrhea, Malnutrition, Weight loss, Skin lesion, Hepatosplenomegaly | Sepsis shock | | Sputum，Blood, Bone marrow | Amphotericin B liposomes, Itraconazole | 22 | 13 | Improvement |
| P13 | 4 | Male | Guangdong | 8 | 0.4 | Fever, Cough, Dyspnea, Malnutrition, Weight loss, Skin lesion, Lymphadenopathy, Hepatosplenomegaly, Ascites | ARDS, HLH, Sepsis, MODS | | Ascites，Blood, Bone marrow | Voriconazole, Itraconazole | 3.5 | 29 | Died |
| P14 | 14 | Male | Guangdong | 366 | 12 | Fever, Cough, Diarrhea, Malnutrition, Weight loss, Hepatosplenomegaly | Sepsis shock | | Blood, Bone marrow | Micafungin, Voriconazole | 26 | 33 | Improvement |
| P15 | 29 | Female | Guangdong | 7 | 0.3 | Fever, Cough, Dyspnea, Malnutrition, Weight loss, Hepatosplenomegaly |  | | Sputum, BALF | Micafungin, Amphotericin B liposomes | 12 | 13 | Improvement |
| P16 | 17 | Male | Yunnan | 8 | 0.4 | Fever, Cough, Diarrhea, Dyspnea, Malnutrition, Weight loss, Lymphadenopathy, Hepatosplenomegaly | ARDS, Sepsis shock | | BALF, Blood, Bone marrow | Amphotericin B liposomes, Itraconazole | 3.5 | 26 | Died |
| P17 | 200 | Male | Hubei | 60 | 2 | Fever, Cough, Malnutrition, Weight loss, Lymphadenopathy, Hepatosplenomegaly | |  | Lymph nodes, BALF | Itraconazole | 20 | 18 | Improvement |
| P18 | 98 | Female | Hunan | 33 | 1 | Fever, Cough, Malnutrition, Weight loss, Hepatosplenomegaly |  | | Lung biopsy | Voriconazole | 26 | 2 | Improvement |
| Abbreviation: ARDS: acute respiratory distress syndrome; MODS: multiple organ dysfunction syndrome; HLH: hemophagocytic lymph histiocytosis; DIC: disseminated intravascular coagulation; BALF: bronchial alveolar lavage fluid | | | | | | | | | | | | | |
