# Supplementary material for: Human inborn errors of immunity underlying Talaromyces marneffei infections: a multicenter, retrospective cohort study
Source: Front Immunol. 2025 Jan 22;16:1492000. doi: 10.3389/fimmu.2025.1492000 (PMC11794527; doi:10.3389/fimmu.2025.1492000)
Supplement: Supplementary file 2 [file Table2.docx]

| **TABLE S2 Peripheral immunologic results of IEIs children with *T. marneffei*** | | | | | | | | | | | |  |
| --- | --- | --- | --- | --- | --- | --- | --- | --- | --- | --- | --- | --- |
| Patients | IgG(g/L) (Normal range) | IgA(g/L) (Normal range) | IgM(g/L) (Normal range) | IgE(IU/ML) (Normal range) | C3(g/L) (Normal range) | C4(g/L) (Normal range) | CD3+4+(cells/ul) (Normal range) | CD3+8+(cells/ul) (Normal range) | CD19+Abs(cells/ul) (Normal range) | CD4/CD8 (Normal range) | NK Abs(cells/ul) (Normal range) | |
| P1 | 15.9 (6.6-10.39) | 0.33 (0.58-1.0) | 1.04 (1.1-1.8) | 1230 (0-60) | 1.03 (0.79-1.52) | 0.32 (0.16-0.38) | 755 (410-1590) | 632 (190-1140) | 306 (90-660) | 1.19 (1.0-2.0) | 91 (90-590) | |
| P2 | 11.09 (7.91-10.07) | 0.93 (0.85-1.71) | 1.96 (1.30-2.26) | 23 (0-60) | 1.012 (0.79-1.52) | 0.334 (0.16-0.38) | 678.67 (410-1590) | 487.22 (190-1140) | 214.18 (90-660) | 1.24 (1.0-2.0) | 143.76 (90-590) | |
| P3 | 7.87 (5.09-10.09) | 0.34 (0.31-0.67) | 1.03 (0.98-1.78) | 30 (0-60) | 1.478 (0.79-1.52) | 0.447 (0.16-0.38) | 20.88 (410-1590) | 32.48 (190-1140) | 753.54 (90-660) | 1.09 (1.0-2.0) | 45.65 (90-590) | |
| P4 | 13.6 (8.0-18) | 1.05 (0.9-4.5) | 1.47 (0.84-1.32) | 1350 (0-60) | 1.474 (0.79-1.52) | 0.431 (0.16-0.38) | 1008.23 (410-1590) | 1225.34 (190-1140) | 366.24 (90-660) | 0.8 (1.0-2.0) | 766.8 (90-590) | |
| P5 | 0.52 (3.52-10.69) | 0.1 (0.13-1.38) | 0.53 (0.38-2.03) | <5 (0-60) | 2.38 (0.51-1.45) | 0.51 (0.08-0.36) | 1746.12 (410-1590) | 431.18 (190-1140) | 2279.28 (90-660) | 4.05 (0.47-2.05) | 161 (90-590) | |
| P6 | <0.33 (3.82-10.58) | 0.17 (0.14-1.14) | 2.65 (0.4-1.28) | <5 (0-60) | 1.48 (0.8-1.5) | 2.65 (0.12-0.4) | 1458.66 (410-1590) | 347.75 (190-1140) | 364.43 (90-660) | 4.19 (0.47-2.05) | 262.96 (90-590) | |
| P7 | 5.37 (3.82-10.58) | <0.07 (0.14-1.14) | 0.33 (0.4-1.28) | <5 (0-60) | 1.29 (0.8-1.5) | 0.63 (0.12-0.4) | 3323.59 (410-1590) | 1350.24 (190-1140) | 1945.34 (90-660) | 2.46 (0.47-2.05) | 479.35 (90-590) | |
| P8 | 0.48 (3.22-7.18) | 0.54 (0.13-0.35) | 0.46 (0.23-0.91) | 15 (0-15) | 0.35 (0.74-1.38) | 0.13 (0.1-0.35) | 2.79 (410-1590) | 16.4 (190-1140) | 76.21 (90-660) | 1.00 (0.47-2.05) | 1.36 (90-590) | |
| P9 | 12.9 (3.82-10.58) | 0.24 (0.14-1.14) | 2.62 (0.4-1.28) | 422 (0-60) | 0.93 (0.8-1.5) | 0.13 (0.12-0.4) | 664.87 (410-1590) | 418.42 (190-1140) | 798.31 (90-660) | 1.59 (0.47-2.05) | 10.91 (90-590) | |
| P10 | 10.1 (6.36-13.24) | 1.07 (0.49-2.29) | 0.61 (0.42-1.46) | 7660 (0-200) | 1.02 (0.8-1.5) | 0.3 (0.12-0.4) | 323.88 (345-2350) | 576.34 (314-2080) | 233.91 (240-1317) | 0.56 (0.9-2.0) | 75.91 (210-1514) | |
| P11 | 3.4 (3.82-10.58) | 0.42 (0.14-1.14) | 1.86 (0.4-1.28) | 46 (0-60) | 0.68 (0.8-1.5) | 0.17 (0.12-0.4) | 703.83 (410-1590) | 250.16 (190-1140) | 810.15 (90-660) | 2.81 (0.47-2.05) | 187.45 (90-590) | |
| P12 | 2.32 (2.86-16.8) | 0.01 (0.19-2.22) | 0.02 (0.43-1.63) | <5 (0-60) | 0.77 (0.8-1.5) | 0.22 (0.11-0.61) | 67 (404-1612) | 184 (220-1129) | 3708 (80-616) | 0.37 (1.0-2.0) | 76 (84-724) | |
| P13 | 2.72 (2.86-16.8) | 0.06 (0.19-2.22) | 0.05 (0.43-1.63) | <5 (0-60) | 1.32 (0.7-2.06) | 0.42 (0.11-0.61) | 6 (404-1612) | 186 (220-1129) | 65 (80-616) | 0.03 (1.0-2.0) | 28 (84-724) | |
| P14 | 11.83 (2.86-16.8) | 0.09 (0.19-2.22) | 0.38 (0.43-1.63) | <5 (0-60) | 1.64 (0.7-2.06) | 0.26 (0.11-0.61) | 1751 (404-1612) | 740 (220-1129) | 438 (80-616) | 2.37 (1.0-2.0) | 56 (84-724) | |
| P15 | 15.03 (2.86-16.8) | 1.29 (0.19-2.22) | 2.15 (0.43-1.63) | 2310 (0-60) | 1.53 (0.7-2.06) | 0.32 (0.11-0.61) | 711 (404-1612) | 282 (220-1129) | 1952 (80-616) | 2.52 (1.0-2.0) | 113 (84-724) | |
| P16 | 1.09 (5.09-10.09) | 0.19 (0.19-2.22) | 1.02 (0.43-1.63) | <5 (0-60) | 1.23 (0.7-2.06) | 0.22 (0.11-0.61) | 121 (404-1612) | 119 (220-1129) | 76 (80-616) | 1.02 (1.0-2.0) | 37 (84-724) | |
| P17 | 14.36 (5.28-21.9) | 3.7 (0.51-2.97) | 1.09 (0.48-2.26) | 15 (0-60) | 1.55 (0.7-2.06) | 0.44 (0.11-0.61) | 187 (404-1612) | 141 (220-1129) | 79 (80-616) | 1.33 (1.0-2.0) | 20 (84-724) | |
| P18 | 22.71 (5.28-21.9) | 1.61 (0.51-2.97) | 1.09 (0.48-2.26) | <5 (0-60) | 1.14 (0.7-2.06) | 0.12 (0.11-0.61) | 756 (404-1612) | 347 (220-1129) | 507 (80-616) | 2.18 (1.0-2.0) | 66 (84-724) | |
|  |  |  |  |  |  |  |  |  |  |  |  | |
