# Supplementary material for: Human inborn errors of immunity underlying Talaromyces marneffei infections: a multicenter, retrospective cohort study
Source: Front Immunol. 2025 Jan 22;16:1492000. doi: 10.3389/fimmu.2025.1492000 (PMC11794527; doi:10.3389/fimmu.2025.1492000)
Supplement: Supplementary file 3 [file Table3.docx]

| **TABLE S3 MAF, CADD score and REVEL for all novel missense variants** | | | | | | |
| --- | --- | --- | --- | --- | --- | --- |
| Patient | Genetic locus | Nucleotide variation | Types of gene mutation | MAF | CADD score | REVEL |
| P10 | *STAT3* | c.1593A>T | Missense mutation | - | 20.2 | 0.751 |
| P11 | *CARD9* | c.1118G>C | Missense mutation | 0.00004542 | 15.73 | 0.181 |
| P15 | *STAT3* | c.115G>A | Missense mutation | - | 33 | 0.827 |
| P16 | *ADA* | c.202T>A | Missense mutation | - | 28 | 0.877 |
| Abbreviation: MAF: Minor Allele Frequency; CADD: Combined Annotation Dependent Depletion; REVEL: Rare Exome Variant Ensemble Learner; STAT: signal transducers and activators of transcription; CARD: Caspase-recruitment domain; ADA: Adenosine deaminase. | | | | | | |
