# Supplementary material for: Human inborn errors of immunity underlying Talaromyces marneffei infections: a multicenter, retrospective cohort study
Source: Front Immunol. 2025 Jan 22;16:1492000. doi: 10.3389/fimmu.2025.1492000 (PMC11794527; doi:10.3389/fimmu.2025.1492000)
Supplement: Supplementary file 4 [file Table4.docx]

| **TABLE S4 NIH SCORE of three patients with novel *STAT3* variants** | | | |
| --- | --- | --- | --- |
| Clinical features | P9 | P10 | P15 |
| IgE score(max IU/ml) | 1 | 10 | 10 |
| Skin abscess(times) | 0 | 0 | 0 |
| Pneumonia(times) | 4 | 4 | 6 |
| Lung anomalies | 8 | 8 | 8 |
| Retained primary teeth | 0 | 0 | 0 |
| Scoliosis | 0 | 2 | 0 |
| Fractures | 0 | 0 | 0 |
| EOS(max ×10^9^/L) | 6 | 6 | 6 |
| Characteristic asymmetric face | 0 | 5 | 2 |
| Newborn rash | 4 | 0 | 4 |
| Eczema | 4 | 4 | 2 |
| URIs/year | 2 | 0 | 2 |
| Candiadias | 4 | 4 | 4 |
| Serious infections | 4 | 4 | 4 |
| Hyperextensibility of joints | 0 | 0 | 0 |
| Lymphoma | 0 | 0 | 0 |
| High palate | 0 | 1 | 1 |
| Age-adjusted | 5 | 0 | 3 |
| **NIH score** | 42 | 48 | 52 |
| Abbreviation: NIH: the National Institutes of Health; STAT: signal transducers and activators of transcription; EOS: eosinophil. | | | |
